# A Novel Method for Preparation of Zn-Doped CuInS_**2**_ Solar Cells and Their Photovoltaic Performance

**DOI:** 10.1155/2013/798713

**Published:** 2013-12-17

**Authors:** Cheng-Hsiung Peng, Chyi-Ching Hwang

**Affiliations:** ^1^Department of Chemical and Materials Engineering, Minghsin University of Science and Technology, Hsinfeng, Hsinchu 304, Taiwan; ^2^Weapon System Center, Chung Cheng Institute of Technology, NDU, Daxi, Taoyuan 335, Taiwan

## Abstract

In this study, a novel method was proposed to synthesize high quality Zn-doped CuInS_2_ nanocrystals under high frequency magnetic field at ambient conditions. The magnetic Zn-doping gave superparamagnetic heating of the resulting nanocrystals via magnetic induction, causing an accelerating growth rate of the doped CuInS_2_ under ambient conditions faster than conventional autoclave synthesis. Shape evolution of the Zn-doped CuInS_2_ nanocrystals from initially spherical to pyramidal, to cubic, and finally to a bar geometry was detected as a function of time of exposure to magnetic induction. These colloidal solvents with different shaped nanocrystals were further used as “nanoink” to fabricate a simple thin film solar device; the best efficiency we obtained of these crystals was 1.01% with a 1.012 **μ**m thickness absorber layer (bar geometry). The efficiency could be promoted to 1.44% after the absorber was thickened to 2.132 **μ**m.

## 1. Introduction

Colloidal semiconductors and metals have been synthesized using coordinating nonaqueous media by manipulating capping ligands, ligand-solvent pairs, reactant concentration, or synthesis temperature [1–5]. CuInS_2_ (termed as CIS hereafter), which is a ternary chalcopyrite compound, has demonstrated more optically and electronically tunable properties than the binary II-VI analogues with the cubic zinc blend structure. CIS has recently been considered to be a promising candidate for photovoltaic applications, owing to its relatively high absorption coefficient and excellent energy matching between its band gap (1.5 eV) and the solar spectrum. CIS nanocrystals have been synthesized by processes such as the elemental solvothermal technique [6, 7], thermolysis [8, 9], the hot injection technique [10, 11], and the single-source precursor route [12, 13]. All of those techniques require high temperature and/or high pressure environments in order to bring the various kinds of species into the desirable crystal form. However, compared with the significant progress in monodisperse binary chalcopyrite colloids, investigation of ternary chalcopyrite colloids has been limited, owing to the lack of suitable synthesis methods. Therefore, the challenge remains in the preparation of monodisperse ternary chalcopyrite colloids with manageable size and shape.

The ternary chalcopyrite semiconductors expressed as I-III-VI_2_ are considered to be a superstructure of the zinc-blende type. In particular, CuAB_2_ (A = Al, Ga, In; B = S, Se) has shown intrinsic p-type conductivity, which suggests that chalcopyrite compounds might be interesting host materials for magnetic doping. CIS nanocrystals have been found to exhibit magnetic properties in a limited number of reports [14, 15]. Taking these findings into consideration, we hypothesized that a new synthesis technology could be advanced by magnetic doping, in which CIS nanocrystal nucleation and growth could be self-manipulated via magnetically induced heating of the developing CIS nanocrystals upon synthesis. This is in contrast to the currently existing time-consuming, cost-ineffective, and eco-unfriendly autoclaving synthesis.

Here, we report a novel methodology for the synthesis of CIS nanocrystals in coordinating solvents without sequential chalcogenide precursor injection, which is achieved by incorporation of magnetic Zn species, that is, diethyldithiocarbamic zinc (DECZn), following a high frequency magnetic field induction (HFMF) at ambient conditions. A series of Zn-doped CIS (termed as Zn-CIS) nanocrystal-based solar cell devices using the nanocrystals with various shapes were also prepared to evaluate their performances.

## 2. Experimental Procedure

### 2.1. Chemicals

Copper (I) chloride (CuCl, 95%, analytical reagent), indium (III) iodide (InCl_3_, 98%, AR), and trioctylphosphine (TOP, 90%, technical grade) were purchased from Sigma-Aldrich Corp.; octadecene (ODE, 90%, technical grade), oleylamine (70%, technical grade), and diethyldithiocarbamic acid zinc salt ([(C_2_H_5_)_2_NCSS]_2_Zn, technical grade) were purchased from Tokyo Chemical Industry Co., Ltd.

### 2.2. Synthesis of Zn-CIS Nanocrystals

Following our previous report [16], 0.5 mmol diethyldithiocarbamic acid zinc salt was dissolved in 6 mL TOP. The solution was diluted with 24 mL ODE to form a clear solution (solution 1). Then, 0.2 mmol CuCl and InCl_3_ wAS dissolved in 6 mL oleylamine at 50°C to form another solution (solution 2). Here, amine coordinates the Cu and In ions to produce amine complexes. These two material solutions were mixed to produce a raw material solution. A small aliquot of raw material solution was put into a test tube and exposed to HFMF. Zn-CIS nanocrystals were formed once the color turns to yellow from turbid; the input power was 90 W.

### 2.3. Characterization

HFMF was set up from power supply, functional generator, amplifier, and cooling water. Similar equipment was also reported in PNAS, vol. 103, 3540–3545 (2006). The strength of the magnetic field depended on the coils. In this study, the coil is 8 loops, frequency is 50 kHz, and the strength of magnetic field (*H*) is 2.5 kA/m. The temperature of HFMF generator was controlled by cycling cooling water at 25°C. X-ray diffraction (XRD) was carried out on a M18XHF diffractometer (MAC Science, Tokyo, Japan) with Cu K*α*_1_ radiation (*λ* = 0.15405 nm) at the operation conditions of 40 kV, 200 mA, 2*θ*: 10°–70°, and scanning rate: 10°/min. Transmission electron microscope (TEM) images were obtained using a JEOL 2100 transmission electron microscope operating at 200 kV. X-ray photoelectron spectroscopy (XPS) measurements were carried out using a Field Emission-Auger Electron Microprobe (Thermo VG Microlab 350) X-ray photoelectron spectrometer using an Mg K*α* X-ray as the excitation source. Then UV-vis, PL emission and excitation (PLE) spectroscopy was applied using a UV-vis spectrophotometer (UV-1600; Agelent 8453) and a spectrofluorometer (FP-6600; Jasco, Inc., Japan).

### 2.4. Fabrication of Zn-CIS Thin Film Solar Cells

The Mo coated soda lime glass substrates used here were fabricated by dc magnetron sputtering at Ar pressures 1.5 mTorr resulting in a 200 nm layer. Deposition of the CIS absorber layer on top of the Mo substrates is used drop casting by the nanoink solution and subsequent thermal treatments to remove the organics and sinter the films under Ar and Se atmospheres at 500°C, respectively. A ~50 nm CdS layer is then deposited by a chemical bath deposition (CBD) technique. The CBD bath contains 183 mL of deionized H_2_O, 25 mL of 0.015 M CdSO_4_ solution, 12.5 mL of 1.5 M thiourea solution, and 31.25 mL of stock NH_4_OH (Aldrich). Next, A ~50 nm high resistance intrinsic zinc oxide (i-ZnO) film capped with a ~300 nm high conductivity indium tin oxide (ITO) layer is deposited by RF magnetron sputtering. The ZnO film is sputtered in a mixture of 10% O_2_ in Ar at sputtering pressure of 10 mTorr without intentional heating. The ITO layer is sputtered with neither O_2_ nor intentional heating at sputtering pressure of 1 mTorr. After sputtering of the oxide layers, the final device is baked in air at 200°C over night.

## 3. Results and Discussion

**Figure 1:**
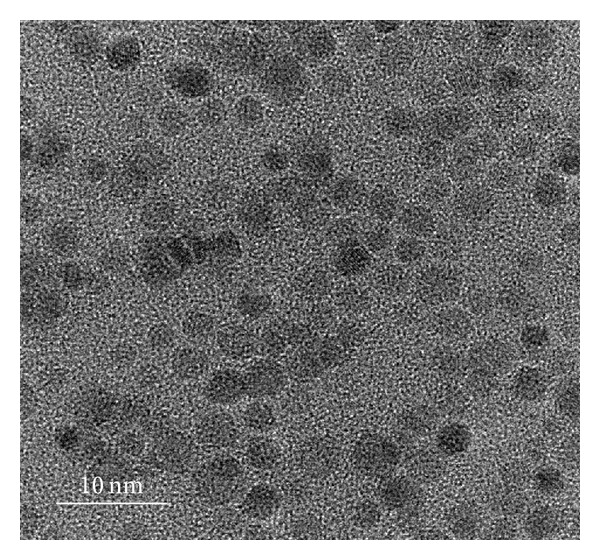
TEM image of Zn-CIS nanoparticles.

**Figure 2:**
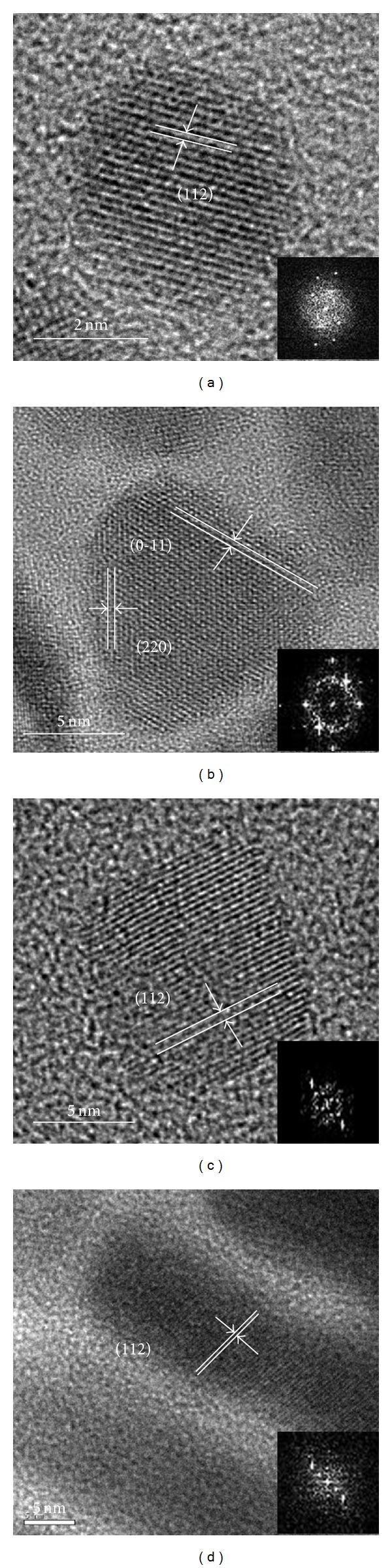
HRTEM images of the Zn-CIS nanocrystals with various geometries; (a) nanoparticle, (b) nanopyramid, (c) nanocube, and (d) nanobar synthesized under magnetic exposure.

Lower-magnification TEM observation was performed to monitor the formation of Zn-CIS nanocrystals. As shown in Figure 1, spherical particles dominantly appeared in the solution in the 60 s sample. The TEM image of these Zn-doped nanocrystals showed narrowly size-distributed nanoparticles, with an average diameter of 3.5 nm. TEM image (Figure 2(a)) further indicated that these nanoparticles were single crystalline and spherical in shape. The size-specific quantum confinement of this size of the Zn-CIS nanocrystals gave a red-color solution. However, after only two minutes of magnetic field exposure, these nanocrystals grew to a size larger than the Wannier-Mott bulk exciton radius (i.e., 4.1 nm for CIS). To identify the crystal growth of the Zn-doped chalcopyrite semiconductor under magnetic exposure, solution samples were taken after magnetic induction for durations of 180, 300, and 420 s, where a steady-state development of the resulting nanocrystals is assumed, albeit unintentionally selected. For the 180-second duration, the colloidal Zn-CIS nanocrystals displayed a rectangular geometry, with a size of 12–15 nm in length (Figure 2(b)). In the TEM images, lattice fringes corresponding to $\left\{\begin{array}{ccc} 0 & -1 & 1 \end{array}\right\}$ and $\left\{\begin{array}{ccc} 2 & 2 & 0 \end{array}\right\}$ CIS planes were predominantly visible. Based on these observations, the structure of the Zn-CIS nanocrystals after 180 s of magnetic induction was confirmed to be trigonal-pyramidal, rather than trigonal-plate, and has been formulated for CuInSe_2_ and CdS. After 300 s of induction, a nanocubic geometry was obtained (from the same batch of solution), as shown in Figure 2(c). The lattice fringes are separated by a distance of 3.1 Å, which corresponds to the $\left\{\begin{array}{ccc} 1 & 1 & 2 \end{array}\right\}$ planes of CIS. The surface tension values of the $\left\{\begin{array}{ccc} 1 & 0 & 0 \end{array}\right\}$, $\left\{\begin{array}{ccc} 0 & 1 & 0 \end{array}\right\}$, and $\left\{\begin{array}{ccc} 0 & 0 & 1 \end{array}\right\}$ planes of nanocubes are very similar, resulting in a similar distance between these three crystallographic faces and Wulff's point. On this basis, a higher average growth rate along those crystallographic directions is expected, resulting in Zn-CIS nanocrystals evolved into rectangular or quasicubic geometry. Nevertheless, after 420 s of magnetic induction, the $\left\{\begin{array}{ccc} 1 & 1 & 2 \end{array}\right\}$ plane of the nanocube showed the fastest growth rate to form a bar-like geometry with an average length of 75.4 nm and width of 18.3 nm (Figure 2(d)).

The molar ratio of Zn : Cu : In : S in the nanocrystals of varying stages of magnetic induction was determined by inductively coupled plasma (ICP) spectroscopy and TEM-EDX analysis. Both data indicated that a relatively uniform compositional evolution of these Zn-CIS nanocrystals can be achieved.

**Figure 3:**
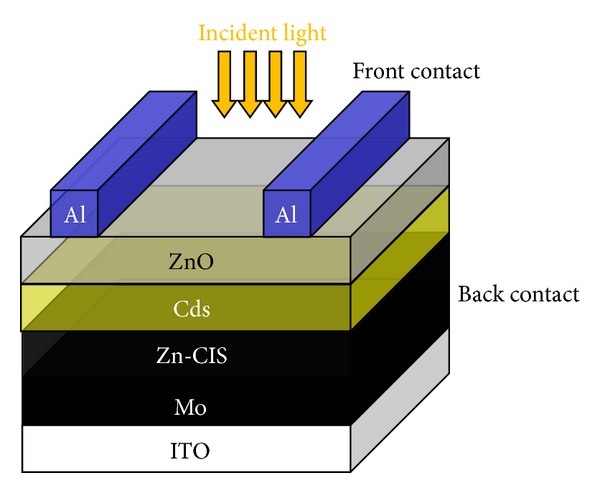
Schematic diagram of the structure of Zn-CIS device.

**Figure 4:**
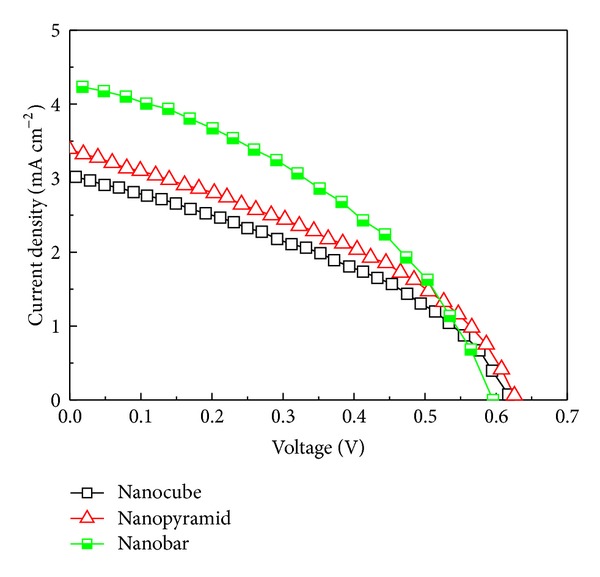
Current-voltage characteristics of Zn-CIS devices with different shape nanocrystal under stimulated AM 1.5 solar illumination (0.1 W/cm^2^) and active area 0.28 cm^2^.

To employ the (Zn-CIS) nanocrystals in solar-cell practical application, we prepared a series of Zn-CIS nanocrystal-based solar cell devices using the nanocrystals of various shapes, to measure the solar-cell parameters. The prototype device is schematically represented in Figure 3, and the current-voltage characteristics of the nanocrystal-based devices are shown in Figure 4. The relevant solar-cell parameters for those three samples are given in Table 1, which include the current density at short circuit (*J*_sc_ in mA cm^−2^), the voltage at open circuit (*V*_oc_ in V), the fill factor (FF), and the efficiency of power conversion (*η* = *J*_sc_ · *V*_oc_ · FF/*P*_in_ with *P*_in_ = 100 mW cm^−2^).

**Figure 5:**
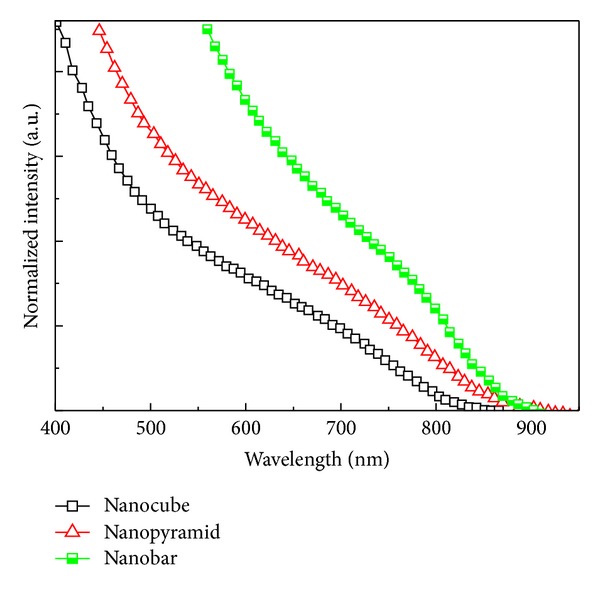
UV-vis absorption spectra of different shape Zn-CIS nanocrystals.

Nanocube-based device displayed the following characteristics: *J*_sc_ = 3.012 mA/cm^2^, *V*_oc_ = 0.61 V, FF = 0.38, and an efficiency of 0.70% on average. The *J*_sc_ values increased from 3.012 to 3.317 mA/cm^2^ at nanopyramid-based device accompanied with increasing efficiency from 0.7 to 0.80%. The maximum value of *J*_sc_ reached 4.21 mA/cm^2^ at nanobar-based device with a power conversion efficiency of 1.01% in this study. This result indicated that the ability of light-absorption of Zn-CIS nanocrystal was enhanced once the crystal size was increased. It is obviously suggested that the increasing efficiency of these devices is related to the broadened absorption wavelength of larger Zn-CIS nanocrystals. The absorption band of Zn-CIS nanocrystals shows a red shift with increasing size, as shown in Figure 5. As can be seen in Table 1, compared to other parameters such as *V*_oc_ and FF, the *J*_sc_ is more determinative for this trend, corresponded to the absorbance spectrum. However, the highest value (4.21 mA/cm^2^) of *J*_sc_ here is still lower than reported (17 mA cm^−2^), which might result from thinner absorber layer. Therefore, we deposited the Zn-CIS layers of different thickness for prototype devices, in order to enhance efficiency of the device by increasing *J*_sc_. (The Zn-CIS nanocrystals used here are a nanobar-like sample.)

**Figure 6:**
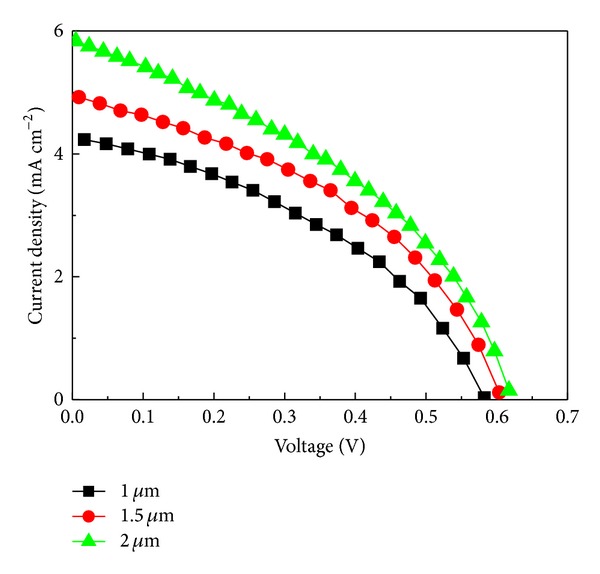
Current-voltage characteristics of Zn-CIS devices with different Zn-CIS film thickness under stimulated AM 1.5 solar illumination (0.1 W/cm^2^) and active area 0.28 cm^2^.

The thickness of Zn-CIS layer can be controlled by varying the nanocrystal concentration in the suspensions.  Figure 6 shows the relationship between absorber layer thickness and the efficiency (the thickness here measured by *α*-step analysis). As summarized in Table 2, after increasing the thickness of Zn-CIS film, the *V*_oc_ value shows a slight increase from 0.59 to 0.62 V which is related to the amount of Zn in the Zn-CIS layer. The amount of Zn in the nanobar-like nanocrystal is lightly more than that in other two kinds of nanocrystals, which might provide explanation to the variation of the *V*_oc_. The FF value shows a decline from 0.41 to 0.39 at 2.132 *μ*m, indicating the increasing charge recombination with thicker Zn-CIS absorber film. The source of charge recombination might have been a result of film cracking, which became more significant for thicker films than for those two thinner ones. The results display a notably systematic trend for *J*_sc_, such that the current density increases significantly from *J*_sc_ = 4.21 mA/cm^2^ at 1.012 *μ*m to *J*_sc_ = 5.871 mA/cm^2^ at 2.132 *μ*m because thicker absorber offers a promotion of light absorption and photocarrier collection. Because the extent of the increase in *J*_sc_ was much greater than the extent of the decrease in FF, the overall efficiency of conversion of photons to current exhibits a systematic increase from *η* = 1.01% at 1.012 *μ*m to *η* = 1.44% at 2.132 *μ*m.

These devices provide a baseline performance and demonstrate as a proof-of-concept that these nanocrystals can be used in PVs. Practical devices, however, require higher efficiencies. There are many ways to promote PV efficiency, including using nanocrystals with shorter chain capping ligands, incorporation of Ga into the films, and using various chemical or thermal treatment of nanocrystal layers to increase their conductivity. New device architectures that are more favorable to using nanocrystal absorber layers and low-temperature manufacturing steps may also provide ways to increase device efficiency and eliminate the need for high temperature processing. These are all important topics for further study.

## 4. Conclusion

In this thesis, we study the synthesis of magnetic-induced synthesized Zn-CIS nanocrystals. All these nanocrystals with variety in shapes are monodispersed and highly crystalline, indicating convenience, rapidity, and novelty of our method. This result demonstrates the fast crystal growth under magnetic-induced heating of such magnetic dopant chalcopyrite semiconductors has potential for energy application. A series of solar cell devices using Zn-CIS nanocrystals of various shapes were fabricated by nanoink process. The best efficiency we obtained is 1.01% for the nanobar-based device, with *J*_sc_ = 4.12 mA/cm^2^, *V*_oc_ = 0.59 V, and FF = 0.41. The thickness of absorber layer in these devices is around 0.983–1.012 *μ*m. The efficiency of performance is promoted to 1.41% after deposition of thicker absorber layer (2.132 *μ*m).

## Figures and Tables

**Table 1:** Photovoltaic performance of the different shape Zn-CIS nanocrystal-based devices under AM 1.5 solar illumination (100 mW/cm^2^) and active area 0.28 cm^2^.

	Nanocube	Nanopyramid	Nanobar
*J*_sc_/mA cm^−2^	3.012	3.317	4.21
*V*_oc_/V	0.61	0.62	0.59
FF	0.38	0.39	0.41
*η* (%)	0.7	0.80	1.01

**Table 2:** Photovoltaic performance of the different thickness of Zn-CIS nanocrystal film devices under AM 1.5 solar illumination (100 mW/cm^2^) and active area 0.28 cm^2^.

Thickness (*μ*m)	1.012	1.594	2.132
*J*_sc_/mA cm^−2^	4.21	4.959	5.871
*V*_oc_/V	0.59	0.61	0.62
FF	0.41	0.41	0.39
*η* (%)	1.01	1.25	1.44
